# Functional Characterization of CYP716 Family P450 Enzymes in Triterpenoid Biosynthesis in Tomato

**DOI:** 10.3389/fpls.2017.00021

**Published:** 2017-01-30

**Authors:** Shuhei Yasumoto, Hikaru Seki, Yuko Shimizu, Ery O. Fukushima, Toshiya Muranaka

**Affiliations:** ^1^Department of Biotechnology, Graduate School of Engineering, Osaka UniversitySuita, Japan; ^2^Center for Open Innovation Research and Education, Graduate School of Engineering, Osaka UniversitySuita, Japan

**Keywords:** tomato, Cytochrome P450 monooxygenase, CYP716 family, C-28 oxidase, C-6β oxidase, triterpenoid biosynthesis

## Abstract

Triterpenoids are a group of structurally diverse specialized metabolites that frequently show useful bioactivities. These chemicals are biosynthesized from the common precursor 2,3-oxidosqualene in plants. The carbon skeletons produced by oxidosqualene cyclase (OSC) are usually modified by cytochrome P450 monooxygenases (P450s) and UDP-dependent glycosyltransferases. These biosynthetic enzymes contribute to the structural diversification of plant triterpenoids. Until now, many P450 enzymes have been characterized as triterpenoid oxidases. Among them, the CYP716 family P450 enzymes, which have been isolated from a wide range of plant families, seem to contribute to the triterpenoid structural diversification. Many CYP716 family P450 enzymes have been characterized as the multifunctional triterpene C-28 oxidases, which oxidize α-amyrin and β-amyrin to the widely distributed triterpenoids ursolic and oleanolic acids, respectively. Tomato (*Solanum lycopersicum*) is one of the most important solanaceous crops in the world. However, little information is known regarding its triterpenoid biosynthesis. To understand the mechanism of triterpenoid biosynthesis in tomato, we focused on the function of CYP716 family enzymes as triterpenoid oxidases. We isolated all six CYP716 family genes from the Micro-Tom cultivar of tomato, and functionally characterized them in the heterologous yeast expression system. The *in vivo* enzymatic assays showed that CYP716A44 and CYP716A46 exhibited the ordinary C-28 oxidation activity against α-amyrin and β-amyrin to produce ursolic and oleanolic acids, respectively. Interestingly, one CYP716E subfamily enzyme, CYP716E26, exhibited the previously unreported C-6β hydroxylation activity against β-amyrin to produce a rare bioactive triterpenoid, daturadiol (olean-12-ene-3β,6β-diol). To determine the roles of the CYP716 family genes in tomato triterpenoid biosynthesis, we analyzed the gene expression and triterpenoid accumulation patterns in different plant tissues by performing the quantitative real-time polymerase chain reaction (qPCR) and gas chromatography-mass spectrometry (GC-MS) analyses, respectively. High levels of the *CYP716A44* gene expression and the accumulation of C-28-oxidized triterpenoids, ursolic acid, and oleanolic acid were observed in the roots, indicating a significant contribution of the *CYP716A44* gene in the triterpenoid biosynthesis in tomato. Thus, our study partially elucidated the mechanism of triterpenoid biosynthesis in tomato, and identified CYP716E26 as a novel C-6β hydroxylase for its subsequent use in the combinatorial biosynthesis of bioactive triterpenoids.

## Introduction

Triterpenoids are structurally diverse plant-specialized metabolites that are often used as pharmaceuticals and cosmetics, among others. Their carbon skeletons are biosynthesized from the common precursor, 2,3-oxidosqualene, by cyclization reaction of oxidosqualene cyclase (OSC). The oxidation and glycosylation of their skeletons by cytochrome P450 monooxygenases (P450s) and UDP-dependent glycosyltransferases contribute to their structural diversities. Until now, many P450 family enzymes involved in triterpenoid biosynthesis have been functionally characterized (reviewed in Seki et al., 2015). Since the initial functional characterization of the *Medicago truncatula* CYP716A12 as a multifunctional enzyme that catalyzes the three-step oxidation at the C-28 position of α-amyrin, β-amyrin, and lupeol to ursolic acid, oleanolic acid, and betulinic acid, respectively (Carelli et al., 2011; Fukushima et al., 2011), many CYP716 family enzymes have been isolated and identified as the triterpenoid-oxidizing enzymes in various plant species. Most of them showed the pentacyclic triterpenoid C-28 oxidation activity, as reported in the *M. truncatula* CYP716A12 enzyme. However, some of them showed relatively different oxidation activities against the triterpenoid skeletons. The *Arabidopsis thaliana* CYP716A2, *Artemisia annua* CYP716A14v2, and *Bupleurum falcatum* CYP716Y1 showed the C-22α, C-3, and C-16α oxidation activities, respectively, against the pentacyclic triterpenoids (Moses et al., 2014, 2015b; Yasumoto et al., 2016). Additionally, the enzymes belonging to the CYP716 family showed oxidation activities not only against the pentacyclic triterpenoids, but also against the dammarane-type triterpenoids. The *Panax ginseng* enzymes CYP716U1 (renamed from CYP716A47; D. Nelson (University of Tennessee) personal communication, Dec. 13 2016) and CYP716S1v2 (renamed from CYP716A53v2 according to the Cytochrome P450 Homepage) have been identified as the dammarene-type triterpenoid C-12 and C-6α oxidases, respectively (Nelson, 2009; Han et al., 2011, 2012). Owing to its divergent origin and diverse triterpenoid oxidation activities, the CYP716 family is considered an important P450 family that contributes to the structural diversification of plant triterpenoids. Tomato (*Solanum lycopersicum*) is one of the most important crops in the world and used as a model plant in the Solanaceae family. Many studies of specialized (secondary) metabolites in tomato are conducted and focused on carotenoids (Fraser et al., 1994; Lois et al., 2000) and steroidal glycoalkaloids (Itkin et al., 2013; Sawai et al., 2014; Thagun et al., 2016). The latter are also biosynthesized from 2,3-oxidosqualene; however, steroidal glycoalkaloids are often excluded from triterpenoids and classified as steroids or sterols. Therefore, information about non-steroidal triterpenoids in tomato is restricted. Triterpenoid profiles in tomato have been analyzed previously (Bauer et al., 2004; Wang et al., 2011). However, oxidized triterpenoids such as ursolic acid and oleanolic acid were not detected except for in a recent report (Kalogeropoulos et al., 2012). In addition, only two *OSCs* were characterized as triterpenoid biosynthetic genes in tomato, and no P450 enzymes were functionally characterized as triterpene oxidases. According to the Cytochrome P450 Homepage (Nelson, 2009), the tomato genome comprises six CYP716 family genes encoding two CYP716A subfamily enzymes (CYP716A44 and CYP716A46), one CYP716C subfamily enzyme (CYP716C6), two CYP716E subfamily [CYP716E25 (previous name CYP716A42), and CYP716E26 (previous name CYP716A43)], and one CYP716H subfamily enzyme (D. Nelson personal communication Dec. 13 2016). In the present study, to determine the mechanism of triterpenoid biosynthesis in tomato, we cloned all six CYP716 family genes from Micro-Tom, a model tomato cultivar, and expressed them in a triterpene-producing yeast strain to determine their oxidation activity. We analyzed the expression levels of the identified triterpenoid biosynthetic genes, and determined the triterpenoid contents in tomato plants. Our study will contribute to the better understanding of triterpenoid biosynthesis in tomato, and provide useful information for the diversification of triterpenoid oxidation reactions catalyzed by the CYP716 family enzymes.

## Materials and methods

### Plasmid construction

The primer sequences used in the present study are listed in Supplementary Table 1. PrimeSTAR Max DNA Polymerase or PrimeSTAR HS DNA Polymerase (TaKaRa Bio, Shiga, Japan) was used according to the manufacturer's protocol. The full-length coding sequences of tomato CYP716A44 and CYP716H1 were amplified from Kazusa Full-length Tomato cDNA clones (clone nos. LEFL3159E12 and LEFL1025BA08) using primer sets 1343/1344 and 1345/1346, respectively. The full-length coding sequences of CYP716E25 (previous name CYP716A42) and CYP716C6 were amplified from the first-strand cDNA prepared from Micro-Tom total RNA extracted from roots with primer sets pY325/pY326 and pY323/pY324, respectively. Each amplicon was cloned into the pENTR-D-TOPO vector (Thermo Fisher Scientific, Waltham, MA, USA) by TOPO reaction. The full-length coding sequences of CYP716E26 (previous name CYP716A43) were amplified from the first-strand cDNA prepared from total RNA extracted from Micro-Tom roots with the primer set pY321/pY322. Further amplification was conducted to adapt the attB sequences to the end of the PCR product using the primer sets pY327/pY328 and pY178/pY179. The final PCR product was cloned into the pDONR/Zeo vector (Thermo Fisher Scientific) using Gateway BP Clonase II Enzyme Mix (Thermo Fisher Scientific). First, since the expression of CYP716A46 in Micro-Tom root was too low to amplify the full-length coding sequence from the first-strand cDNA, the full-length genomic sequence from start codon to stop codon was amplified using Micro-Tom genomic DNA with the primer set pY319/pY320. Genomic *CYP716A46* was cloned into pCR4-Blunt-TOPO vector (Thermo Fisher Scientific). The resulting plasmid DNA was used as a template for amplification of the 1st, 2nd, and 3rd exons of CYP716A46 with adapter primer sets pY319/pY337, pY338/pY339, and pY340/pY320, respectively. The 1st, 2nd, and 3rd exons were joined by PCR reactions. Further amplification was conducted to add the attB sequences to the end of the PCR product using primer sets pY341/pY342 and pY178/pY179. The final PCR product was cloned into the pDONR/Zeo vector (Thermo Fisher Scientific) using Gateway BP Clonase II Enzyme Mix (Thermo Fisher Scientific). The full-length coding sequence in each entry clone was confirmed by Sanger sequencing.

To construct the yeast expression vectors pYES-DEST52-CYP716A44, pELC-CYP716A44, and pESC-HIS-CYP716A44, cDNA of CYP716A44 in entry vector was transferred into pYES-DEST52 (Thermo Fisher Scientific), pELC-GW, and pESC-HIS-GW vectors (Yasumoto et al., 2016) using Gateway LR Clonase II Enzyme Mix (Thermo Fisher Scientific). The same method was applied for the CYP716A46, CYP716C6, CYP716E25, CYP716E26, and CYP716H1 yeast expression vectors, pYES-DEST52-CYP716A46, pELC-CYP716A46, pESC-HIS-CYP716A46, pYES-DEST52-CYP716C6, pELC-CYP716C6, pESC-HIS-CYP716C6, pYES-DEST52-CYP716E25, pELC-CYP716E25, pESC-HIS-CYP716E25, pYES-DEST52-CYP716E26, pELC-CYP716E26, pESC-HIS-CYP716E26, pYES-DEST52-CYP716H1, pELC-CYP716H1, and pESC-HIS-CYP716H1.

### *In vivo* enzymatic analysis

*In vivo* enzymatic analysis was performed as reported previously (Yasumoto et al., 2016). The budding yeast (*Saccharomyces cerevisiae* INV*Sc*1, Thermo Fisher Scientific) harboring pYES-ADH-aAS and pYES3-ADH-bAS was used as α-amyrin and β-amyrin producing yeast strains (Fukushima et al., 2011). Three kinds of yeast expression vectors for CYP716A44 (pELC-CYP716A44, pYES-DEST52-CYP716A44, and pESC-HIS-CYP716A44) were transformed into yeast strains using a Frozen-EZ Yeast Transformation II Kit (Zymo Research, Irvine, CA, USA). Tranformants were selected on SD (-Trp, -Leu, -Ura, and -His) plates to generate *CYP716A44* expressing yeast strains, such as SY52 and SY57. The same strategy was conducted to generate *CYP716A46, CYP716C6, CYP716E25, CYP716E26*, and *CYP716H1* expressing yeast strains. Each of the transformed yeast strains is listed in Supplementary Table 2. Each glycerol stock was inoculated into 2 mL SD medium containing 2% glucose and cultured at 30°C overnight at 200 rpm. A 10mL new SD medium was added to the culture overnight, subsequently cultured at 30°C for an additional night, and shaken at 200 rpm. Yeast cells were collected by centrifugation, resuspended in 10 mL SD medium containing 2% galactose to induce CYP716 expression, and cultured at 30°C for 2 days at 200 rpm. Yeast cultures were then stored at −80°C until extraction. Yeast cultures were extracted three times with 6 mL ethyl acetate. The extracts were evaporated, and the remaining residues were dissolved in 1mL chloroform:methanol (1:1). The samples were stored at −30°C until use. A 50-μL of the sample and standard solutions (α-amyrin, uvaol, ursolic acid, β-amyrin, erythrodiol, and oleanolic acid were purchased from Sigma-Aldrich or EXTRASYNTHESE, 10 mg/L in methanol) was transferred into vial inserts and evaporated. The sample was derivatized with 50 μL N-methyl-N-(trimethylsilyl) trifluoroacetamide at 80°C for 20 min. Gas chromatography-mass spectrometry (GC-MS) analysis was performed using a 5977A GC-MS system (Agilent Technologies, Santa Clara, CA, USA) with a DB-1 ms capillary column. GC-MS conditions were reproduced from a previous study (Yasumoto et al., 2016).

### Structural determination of unknown product in β-amyrin synthase/CYP716E26 co-expressing yeast

Yeast strain SY56 was cultured in 6 L SD medium containing 2% galactose. Yeast cells were collected by centrifugation, incubated with 720 mL methanol and 180 mL 40% KOH at 80°C, and extracted with 900 mL n-hexane three times. The extracts were evaporated, and the remaining residues were applied to column chromatography on silica gel (60 N 40–50 μm) in a hexane:ethyl acetate gradient. After purification, 10 mg of an unknown compound was obtained. Nuclear magnetic resonance (NMR) spectra (^1^H and ^13^C) of purified compound were reported on a JEOL-ECS400 system (400 MHz; JEOL, Tokyo, Japan) in CDCl_3_. Tetramethylsilane was used as an internal standard.

### Quantitative real-time polymerase chain reaction (qPCR) analysis

Total RNA was extracted from leaves, stems, flowers, and roots of tomato cv. Micro-Tom grown on soil using RNAiso Plus (TaKaRa Bio). After digestion of the contaminated genomic DNA by recombinant DNase (TaKaRa Bio), total RNA was purified using an RNeasy Plant Mini Kit (Qiagen, USA). The first-strand cDNA was synthesized from purified total RNA using PrimeScript RT Master Mix (Perfect Real Time) (TaKaRa Bio). qPCR was performed using LightCycler Nano System (Roche, Germany) and FastStart Essential DNA Green Master (Roche) with synthesized cDNA samples. The primer sequences used in qPCR were listed in Supplementary Table 1. The relative expression levels of *CYP716A44, CYP716A46, CYP716C6, CYP716E25, CYP716E26, CYP716H1, TTS1*, and *TTS2* in each sample were calculated according to a previously described method (Pfaffl, 2001). Actin and leaf were used as reference gene and control sample, respectively.

### Triterpenoid analysis in tomato

The leaves, flowers, stems, and roots were harvested from tomato cv. Micro-Tom plants grown on soil, lyophilized, and then crushed to powder by using a multibead shocker (Yasui Kikai, Japan). A 0.1 g of dry materials was extracted three times with 5 mL methanol:chloroform (1:1). After drying by centrifugal evaporator, samples were saponified with 3 mL methanol and 1 mL 40% KOH solution at 80°C for 1 h and extracted with 4 mL n-hexane three times. HCL (1 mL) was added to the remaining solutions and extracted with 4 mL n-hexane three times. The extracts were separately evaporated, and the remaining residues were dissolved in 1 mL methanol:chloroform (1:1). A 50 μL of the sample and standard solutions was transferred into vial inserts and evaporated. The remaining residues were derivatized with 50 μL N-methyl-N-(trimethylsilyl) trifluoroacetamide at 80°C for 20–30 min. GC-MS analysis was performed as mentioned in *in vivo* enzymatic assay. Additionally, a selective ion monitoring (SIM) GC-MS (*m/z* = 203, 218, 320) was also conducted to detect low levels of ursolic and oleanolic acids.

### Phylogenetic tree analysis

The full-length protein sequences of all P450 enzymes, which were functionally characterized as triterpenoid oxidases, were collected from GenBank and aligned using MUSCLE (Edgar, 2004). A rooted maximum likelihood tree was generated using the LG+G+I substitution model by MEGA6 software (Tamura et al., 2013) with bootstrapping for 1000 replicates. The protein sequences in FASTA format were listed in Supplementary Data sheet 1.

## Results

### Cloning of tomato CYP716 family genes

**Figure 1:**
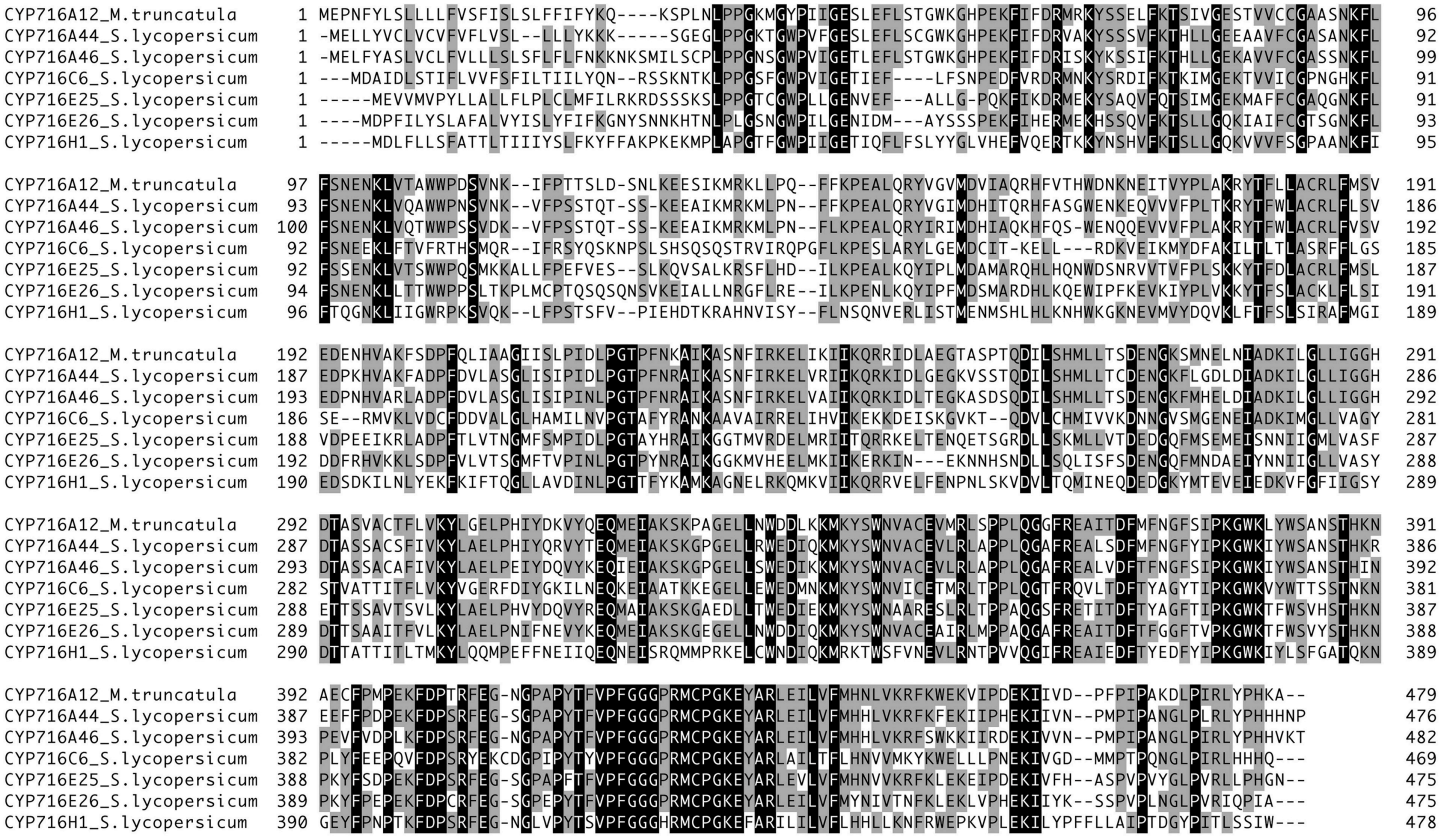
**Multiple alignment of tomato CYP716 family proteins**. Multiple alignment was generated using GENETYX-MAC version 17. Identical amino acids are indicated by dark shading.

The tomato genome encodes six CYP716 family enzymes, CYP716A44 (Solyc05g021390), CYP716A46 (Solyc07g042880), CYP716C6 (Solyc02g069600), CYP716E25 (Solyc06g065420), CYP716E26 (Solyc06g065430), and CYP716H1 (Solyc11g056670), when excluding predicted pseudogenes for CYP716A45P, CYP716H2P, CYP716Q1P, and CYP716Q2P (CYP716A42 and CYP716A43 have been renamed as CYP716E25 and CYP716E26, respectively, D. Nelson personal communication, Dec. 13 2016). We cloned three of the CYP716 family full-length coding sequences [registered in GenBank as predicted genes in cv. Heinz 1706; accession numbers: XM_004233140 (CYP716C6), XM_004242183 (CYP716E25), and XM_004241773 (CYP716E26)] from the first-strand cDNA prepared from Micro-Tom and two others [registered in GenBank as Micro-Tom cDNA clones; accession numbers: AK329870 (CYP716A44) and AK321479 (CYP716H1)] from Kazusa full-length tomato cDNA clones (Figure 1). In the present study, we could not amplify the full-length coding sequence for CYP716A46 by reverse transcription-PCR using the first-strand cDNA prepared from Micro-Tom flowers. Since *CYP716A46* expression is strictly limited in unopened flower buds based on the expression profile in the tomato eFP Browser (http://bar.utoronto.ca/efp_tomato/cgi-bin/efpWeb.cgi), we are possibly not be able to amplify the full-length coding sequence, even if it were expressed in plants. Therefore, we artificially generated the full-length coding sequence for CYP716A46 (registered in GenBank as a predicted gene in cv. Heinz 1706; accession number: XM_004243858) by connecting all exon sequences as described in the Material and Methods Section.

### *In vivo* enzymatic analysis

**Figure 2:**
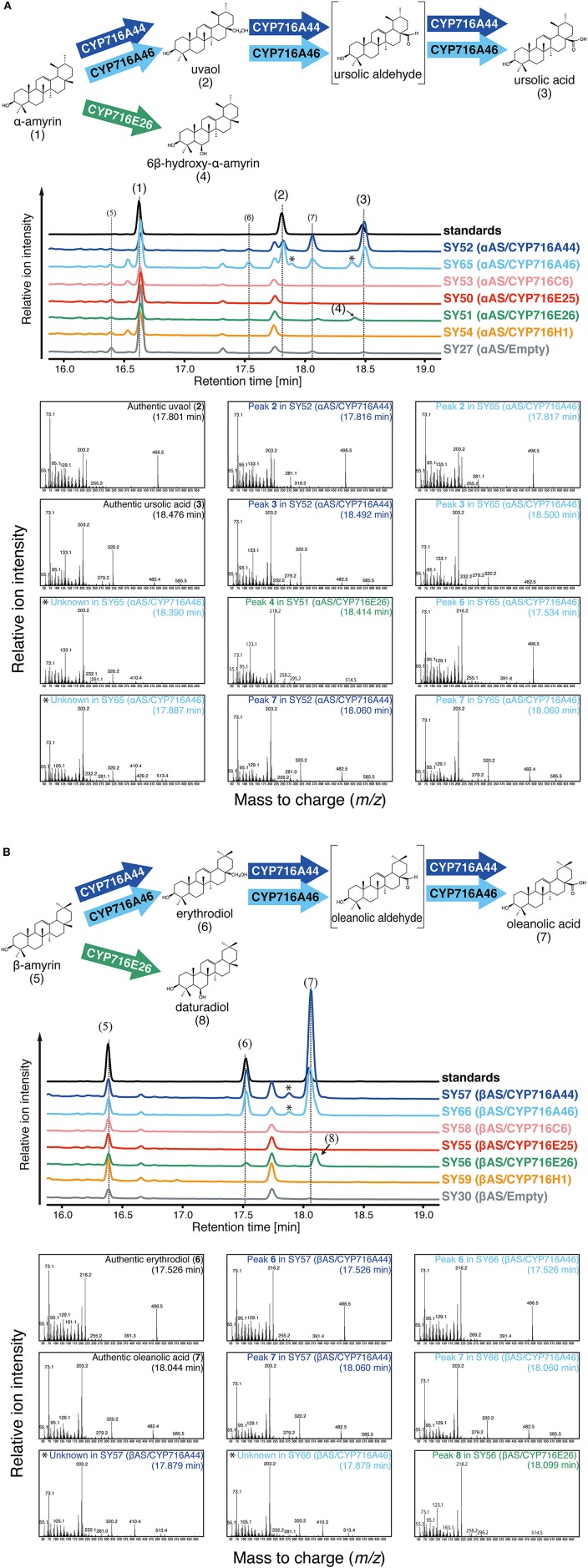
*****In vivo*** enzymatic activity assay in yeast**. The relative intensity of total ion chromatograms (TICs) of yeast extracts and oxidation reactions catalyzed by tomato CYP716 enzymes. **(A)** αAS/CYP716 expressing yeast strains. Mixture of α-amyrin **(1)**, uvaol **(2)**, and ursolic acid **(3)** was used as standard sample. **(B)** βAS/CYP716 expressing yeast strains. Mixture of β-amyrin **(5)**, erythrodiol **(6)**, and oleanolic acid **(7)** was used as standard sample. TICs corresponding to standard, CYP716A44 expressing yeast (SY52 and SY57), CYP716A46 expressing yeast (SY65 and SY66), CYP716C6 expressing yeast (SY53 and SY58), CYP716E25 expressing yeast (SY50 and SY55), CYP716E26 expressing yeast (SY51 and SY56), CYP716H1 expressing yeast (SY54 and SY59), and empty vector containing yeast (SY27 and SY30) are indicated in black, blue, light blue, pink, red, green, orange, and gray color, respectively. All samples were analyzed after TMS derivatization. Major peaks corresponding to the reaction products of P450s are numbered. Oxidation reactions catalyzed by each CYP716 enzyme are indicated by arrows. The predicted chemical structures of the product are shown in parentheses. The peaks likely corresponding to the partially TMS-derivatized (with one TMS molecule) ursolic and oleanolic acids are indicated by asterisks. The mass fragmentation patterns with retention times are also shown below the TICs. The mass fragmentation patterns obtained from authentic standards, CYP716A44, CYP716A46, and CYP716E26 expressing yeast strains are indicated by black, green, blue, and light blue letters, respectively.

The tomato CYP716 family enzymes were heterologously co-expressed with *Olea europaea* α-amyrin synthase (αAS) or *Lotus japonicus* β-amyrin synthase (βAS) in yeast. In the present study, we could not detect clear oxidation activities of CYP716C6, CYP716E25, and CYP716H1 against α-amyrin and β-amyrin in yeast expression system. In αAS/CYP716A44 and αAS/CYP716A46 co-expressing yeast strains (SY52 and SY65), uvaol **(2)** and ursolic acid **(3)** were detected as C-28 oxidation product of α-amyrin **(1)** (Figure 2A). Since the αAS enzyme used in the present study produced not only α-amyrin, but also β-amyrin (Saimaru et al., 2007), the β-amyrin-oxidized triterpenoids could also be detected in the αAS/CYP716A co-expressing yeast strains as minor peaks (Figure 2A). Further, in βAS/CYP716A44 and βAS/CYP716A46 co-expressing yeast strains (SY57 and SY66), erythrodiol **(6)** and oleanolic acid **(7)** were detected as C-28 oxidation product of β-amyrin **(5)** (Figure 2B). Interestingly, an unknown compound **(4)** was detected in αAS/CYP716E26 co-expressing yeast strain (SY51), and other unknown compound **(8)** was also detected in βAS/CYP716E26 co-expressing yeast strain (SY56) (Figure 2). Although, the retention times of those compounds were closed to ursolic acid and oleanolic acid, their mass fragmentation patterns were completely different from those C-28 oxidized triterpenoids (Figure 2). To reveal the oxidation activity of CYP716E26, we conducted a large-scale culture of yeast strain SY56, purified the unknown compound **8**, and determined the structure by NMR analysis (Supplementary Figure 1). As a result, the chemical shift of compound **8** matched well with that of 6β-hydroxy-β-amyrin (olean-12-ene-3β,6β-diol, daturadiol) (Araújo and Chaves, 2005). Thus, we concluded that compound **8** corresponded to daturadiol (Supplementary Table 3). Since the mass spectra of compound **4** were similar to that of daturadiol (Figure 2), and CYP716E26 exhibited 6β-hydroxylation activity against β-amyrin, we hypothesized that CYP716E26 also displays C-6β-hydroxylation activity against α-amyrin. Thus, we predicted that compound **4** might correspond to 6β-hydroxy-α-amyrin (ursan-12-ene-3β,6β-diol). We detected unknown peaks in CYP716A44- or CYP716A46-expressing yeast strains at retention times 17.9 and 18.4 min. Since their mass spectra were similar to those of the ursolic or oleanolic acids [with two trimethylsilyl (TMS) molecules], except for *m/z* = 410 and 513 (482 and 585 in ursolic and oleanolic acids, respectively) and the difference of *m/z* values corresponding to one TMS molecule (*m/z* = 72), these two peaks might correspond to the partially TMS-derivatized (with one TMS molecule) ursolic and oleanolic acids, respectively (Figure 2).

### Expression of triterpenoid biosynthetic genes in tomato

**Figure 3:**
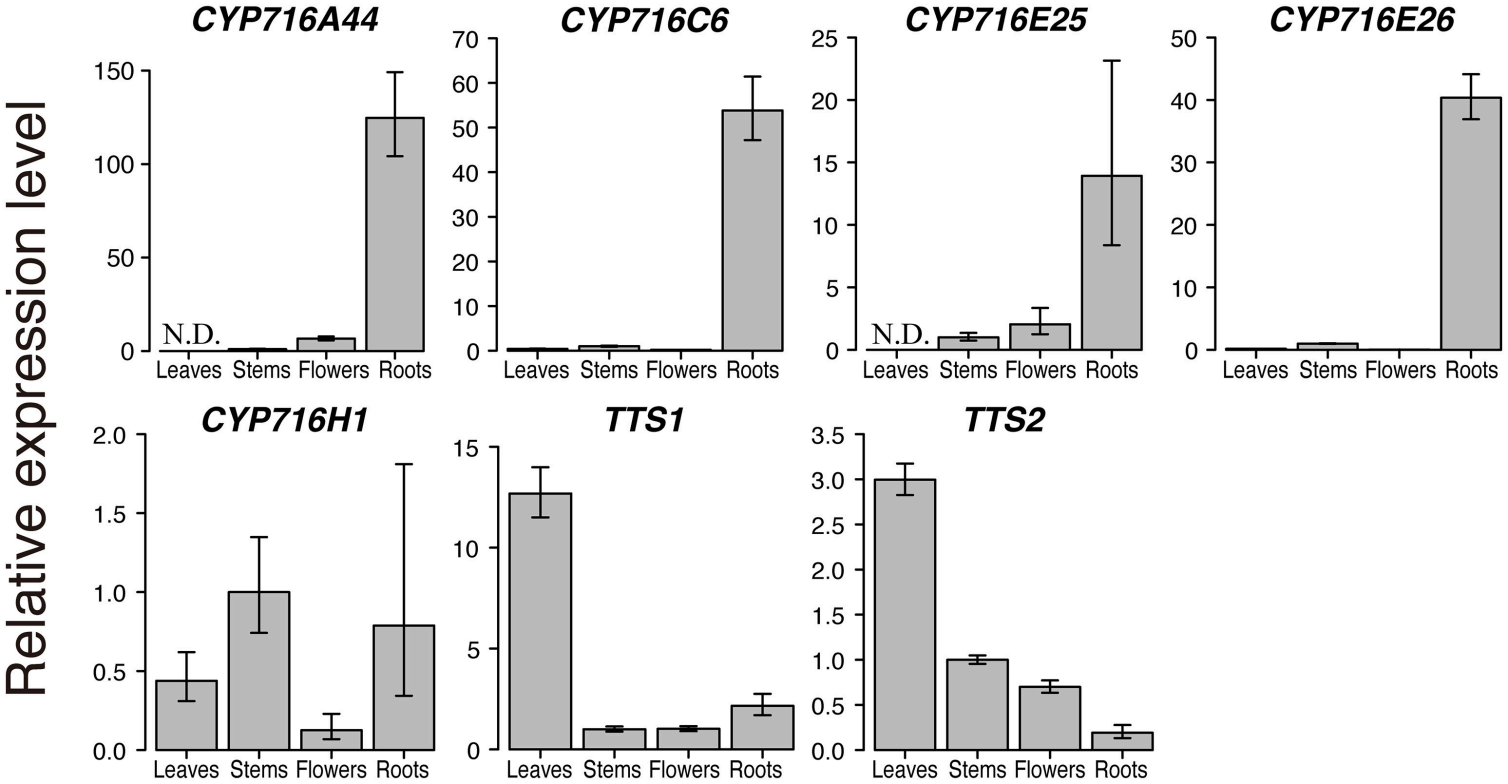
**Quantitative real-time polymerase chain reaction (qPCR) analysis of triterpenoid biosynthetic genes in tomato**. qPCR analysis of the six CYP716 family and two OSC genes, *TTS1* and *TTS2*, in leaves, stems, flowers, and roots of tomato cultivar Micro-Tom. The relative expression levels represent the means, and the vertical bars indicate the standard deviation calculated from technical quadruplicate experiments. N.D. means not detected, and no amplification is observed in the sample. Since the amplification of *CYP716A46* is observed only in flowers, the relative expression level of *CYP716A46* is not shown in this figure.

After the enzymatic assay in yeast expression system, we confirmed the expression levels of CYP716 family genes in tomato leaves, stems, flowers, and roots by qPCR. We included the two *OSCs, TTS1* encoding product specific β-amyrin synthase and *TTS2* encoding multifunctional OSC producing δ-amyrin as a major product with six other products, identified previously in tomato triterpenoid biosynthesis pathway (Wang et al., 2011). *CYP716A44* and *CYP716E26*, which showed C-28 and C-6β oxidizing activities against ursane and oleanane skeletons, were specifically expressed in roots (Figure 3). *CYP716C6* and *CYP716E25*, which showed no oxidation activity in yeast expression system, were also specifically expressed in roots. In contrast, *CYP716H1* was expressed in not only roots but also leaves, stems, and flowers (Figure 3). On the other hand, less *CYP716A46* transcript was detected only in flower sample (data not shown). Interestingly, expression levels of *TTS1* and *TTS2* in roots were lower than those in leaves, stems, and flowers.

### Triterpenoids in tomato

**Figure 4:**
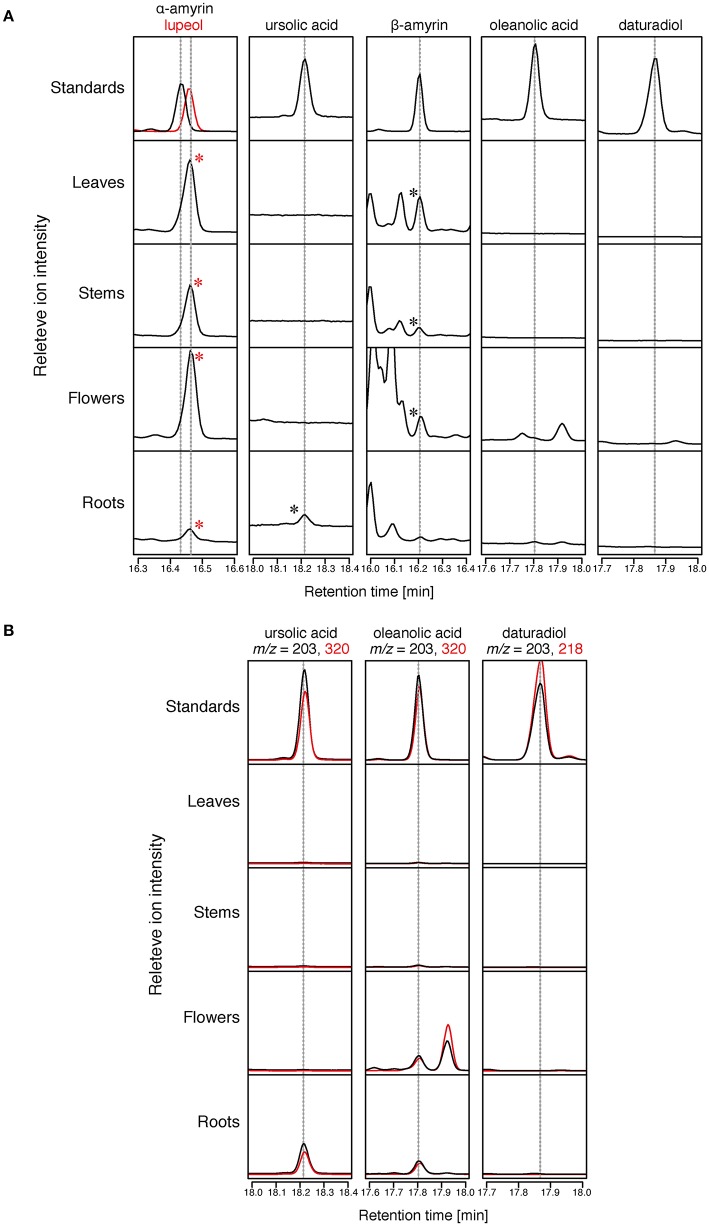
**Triterpenoids distribution in Micro-Tom**. The relative intensity of GC-MS chromatograms of extracts of Micro-Tom leaf, stem, flower, and root. All samples were analyzed after TMS derivatization. **(A)** Total ion chromatograms (TICs) of tomato extracts at retention times of α-amyrin, lupeol, ursolic acid, β-amyrin, oleanolic acid, and daturadiol. The peaks identified by comparing the retention time and mass fragmentation patterns with those of standards are marked with asterisks. Mass fragmentation patterns are shown in Supplementary Figure 2. **(B)** Selective ion monitoring (SIM) chromatograms of tomato extracts at retention times of ursolic acid, oleanolic acid, and daturadiol.

To confirm the distribution of triterpenoid aglycones in the Micro-Tom cultivar of tomato, we saponified (performed alkaline hydrolysis) the extracts of tomato leaves, stems, flowers, and roots, and then analyzed them by GC-MS. The triterpenoid aglycones could be released from the triterpenoid glycosides through the saponification treatment; thus, some of the triterpenoids detected in GC-MS analysis might have originated from the triterpenoid glycosides. Since δ-amyrin, which is reported as a major triterpenoid in Micro-Tom (Wang et al., 2011), was not commercially available, we analyzed only α-amyrin, β-amyrin, ursolic acid, oleanolic acid, and daturadiol (isolated in the present study) by comparing the retention times (RTs) and mass fragmentation patters with those of the authentic standards. β-Amyrin was detected in leaf, stem, and flower samples (Figure 4). In contrast, α-amyrin was not detected in any sample. However, a peak (RT = 16.46 min) close to α-amyrin (RT = 16.44 min) was found in all samples. Since the mass fragmentation pattern of the peak was similar to that of lupeol **(9)**, we additionally analyzed the authentic lupeol and identify that peak as lupeol **(9)** (Figure 4, Supplementary Figure 2). In total ion chromatogram, ursolic acid was detected in roots, and oleanolic acid was not detected in any sample (Figure 4A). However, in SIM chromatogram at *m/z* = 203 and 320, the peaks corresponding to ursolic acid and oleanolic acid were present in roots, and only a peak corresponding to oleanolic acid was detected in flowers (Figure 4B). No peak corresponding to daturadiol could be detected.

## Discussion

### Enzymatic activities of tomato CYP716 family enzymes

**Figure 5:**
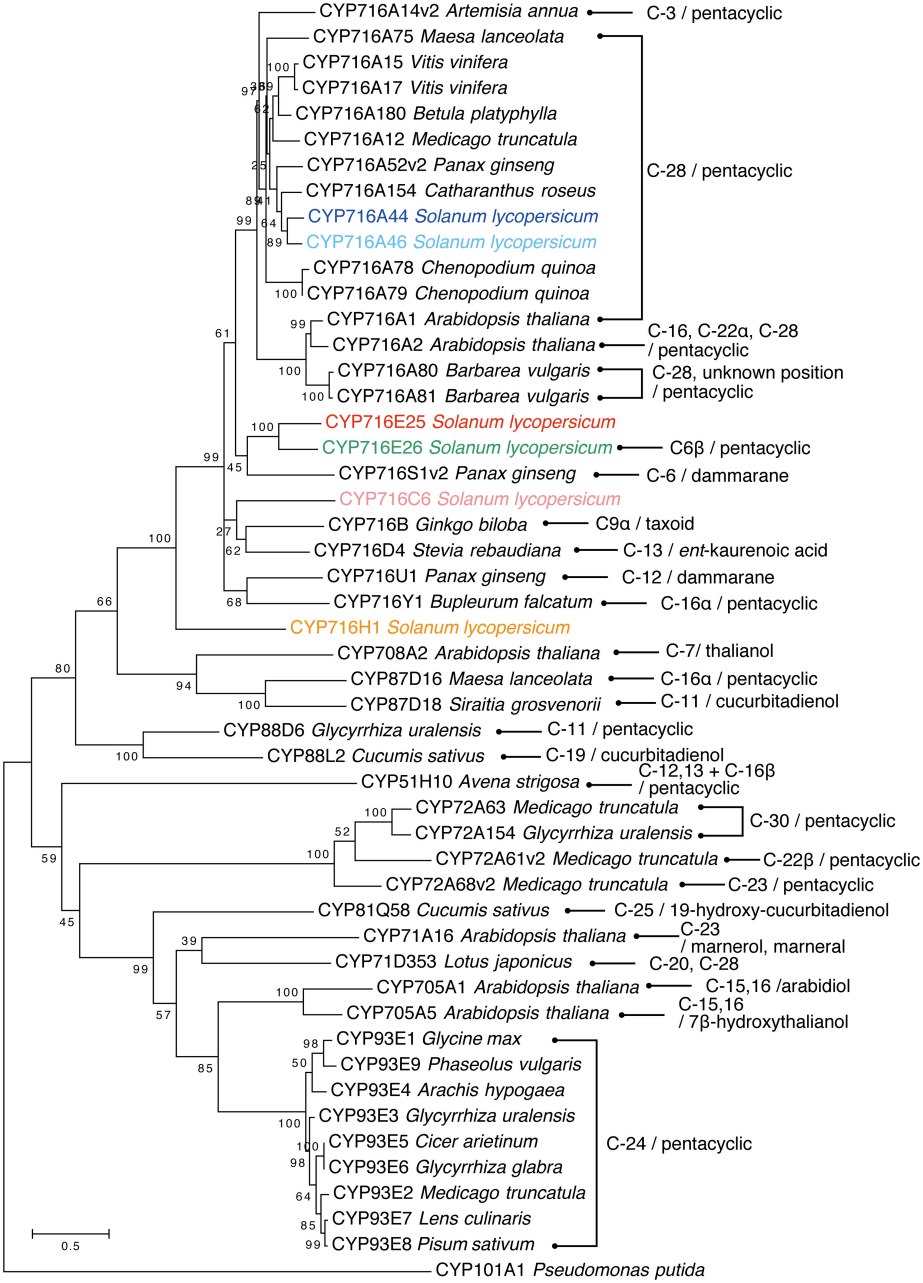
**Phylogenetic tree of triterpenoid oxidases**. The rooted maximum likelihood tree was generated using tomato six CYP716 family enzymes and P450s, which were characterized as triterpenoid oxidases by MUSCLE and MEGA6 software. The oxidation positions on triterpenoid and substrates are indicated (pentacyclic means α-amyrin, β-amyrin, or lupeol skeletons). *Pseudomonas putida* CYP101A1 is used as outgroup. Based on our personal communication with Prof. David Nelson, the enzymes CYP716AL1, CYP716A42, CYP716A43, CYP716A47, and CYP716A53v2 have been renamed as CYP716A154, CYP716E25, CYP716E26, CYP716U1, and CYP716S1v2, respectively.

In the present study, all six *CYP716* family genes (except pseudogenes) annotated from the genome sequence were cloned from tomato cv. Micro-Tom and functionally tested in a yeast expression system. Both of the tested CYP716A subfamily enzymes, CYP716A44 and CYP716A46, showed the ordinary C-28 oxidation activity against the simple triterpene skeletons, such as α-amyrin and β-amyrin (Figure 2). This activity has been previously reported in many CYP716A subfamily enzymes, such as the *M. truncatula* CYP716A12 (Fukushima et al., 2011), *Catharanthus roseus* CYP716A154 (renamed from CYP716AL1, D. Nelson personal communication, Dec. 13 2016; Huang et al., 2012), *P. ginseng* CYP716A52v2 (Han et al., 2013), *Maesa lanceolata* CYP716A75 (Moses et al., 2015a), *Barbarea vulgaris* CYP716A80 (Khakimov et al., 2015), and *A. thaliana* CYP716A1 (Yasumoto et al., 2016). According to the phylogenetic analysis, both CYP716A44 and CYP716A46, which showed 81.6% sequence identity with each other, were clustered together with the previously reported C-28 oxidases (Figure 5).

One of the two tested CYP716E subfamily enzymes, CYP716E26 showed extraordinary C-6β-hydroxylation activity against β-amyrin, and produced daturadiol as a reaction product (Figure 2). This rare triterpenoid was previously isolated from seeds of *Datura innoxia* (Solanaceae; Kocor et al., 1973) and *Datura stramonium* (Itoh et al., 1977) and leaves of *Terminalia brasiliensis* (Combretaceae; Araújo and Chaves, 2005). Additionally, Pei and co-workers isolated cytotoxic triterpenoids including daturadiol from the leaves of *Vernicia fordii* (Euphorbiaceae) and found that daturadiol displayed moderate cytotoxic activity against human cancer cell lines (Pei et al., 2013). Since the genetic information of those plants is not available, it is difficult to identify daturadiol biosynthetic enzymes from them. Our findings provide a method for the production of a rare and bioactive triterpenoid, daturadiol, in transgenic yeast. This is the first report on the functional characterization of CYP716 family enzymes isolated from a solanaceous plant and on an enzyme that oxidizes the C-6β position of triterpene skeleton. This finding contributes to the enrichment of the enzymes useful for the combinatorial biosynthesis of bioactive triterpenoids, and indicates that the enzymes required for the combinatorial triterpenoid biosynthesis could be cloned not only from the medicinal plants, but also from the common crops such as tomato.

In this time, we could not find any detectable oxidation activity of CYP716C6, CYP716E25, and CYP716H1 against α-amyrin and β-amyrin in our yeast expression system. Since some P450 enzymes already require oxidized triterpene backbones as substrates, we cannot exclude the possibility that those enzymes may be involved in triterpenoid biosynthesis. For example, *M. truncatula* CYP72A61v2 and CYP72A68v2 showed C-22β and C-23 oxidation activities against 24-hydroxy-β-amyrin and oleanolic acid, respectively. However, they did not catalyze the oxidation on β-amyrin (Fukushima et al., 2013). *Cucumis sativus* CYP81Q58 did not show oxidation activity against cucurbitadienol, the precursor of bioactive triterpenoid, cucurbitacin C. However, it produced 19,25-dihydroxy-cucurbitadienol when co-expressed with cucurbitadienol C-19 hydroxylase, *C. sativus* CYP88L2 in heterologous yeast expression system, and was identified as a biosynthetic enzyme involved in cucurbitacin C biosynthesis in *C. sativus* (Shang et al., 2014). In the case of tomato, CYP716C6 and CYP716E25 seem to work together with CYP716A44 and/or CYP716E26, because these CYP716 family genes are mainly expressed in the roots. Another possible reason to explain why they did not show the oxidation activity is that they use a different kind of triterpenoid backbone as their substrate. In the present study, we used the common pentacyclic triterpenoids, α-amyrin and β-amyrin, as substrates for the CYP716 family enzymes in tomato. However, some CYP716 family enzymes have been reported to oxidize the tetracyclic triterpenoids. The *A. thaliana* CYP716A1 and *P. ginseng* CYP716U1, and CYP716S1v2 can oxidize the tetracyclic triterpenoids tirucalla-7,24-diene-3β-ol, dammarenediol-II, and protopanaxadiol, respectively (Han et al., 2011, 2012; Boutanaev et al., 2015). This kind of unusual tetracyclic triterpenoids could also act as the potential substrates for tomato CYP716C6, CYP716E25, and CYP716H1. Until now, many gene clusters for the plant specialized metabolisms have been reported (reviewed in Nützmann and Osbourn, 2014). For example, the gene cluster in oat (*Avena* spp.) contains five genes encoding an OSC (β-amyrin synthase), a P450 (CYP51H10) to modify the triterpene scaffold, a UDP-dependent glycosyltransferase, and two other enzymes for the biosynthesis of triterpenoid saponin, avenacin (Nützmann and Osbourn, 2014). Therefore, we tried to find OSC gene(s) that could be clustered with CYP716 family genes so as to predict the potential triterpenoid substrates for the CYP716 family enzymes; however, we could not find any OSC genes near the CYP716 family genes in the tomato genome browser (data not shown). Another possibility is that they might play roles in diterpene biosynthesis; some CYP716 family enzymes except from CYP716A, CYP716E, and CYP716Y subfamilies were characterized as diterpenoid oxidases. For example, *Gingko biloba* CYP716B was functionally characterized as taxoid 9α hydroxylase (Zhang et al., 2014), and *Stevia rebaudiana* CYP716D4 was speculated to function as *ent*-kaurenoic acid 13-hydroxylase (detailed information about this unclear enzyme is available in Ceunen and Geuns, 2013). Thus, to reveal the roles of CYP716C6, CYP716E25, and CYP716H1 in tomato, further analyses are required.

### Phylogenetic analysis of triterpenoid oxidases

Until now, more than 40 P450 enzymes were functionally characterized as triterpenoid oxidases and half of them were classified as CYP716 family (Figure 5). C-28 oxidation of pentacyclic triterpenoids is the predominant reaction catalyzed by CYP716 family enzymes (Figure 5). In the present study, we revealed that CYP716A44 and CYP716A46 also have C-28 oxidation activity, and they clustered with previously characterized C-28 oxidases, such as *C. roseus* CYP716A154 (CYP716AL1) (Huang et al., 2012), *P. ginseng* CYP716A52v2 (Han et al., 2013), *Chenopodium quinoa* CYP716A78 and CYP716A79 (Fiallos-Jurado et al., 2016), *M. truncatula* CYP716A12 (Carelli et al., 2011; Fukushima et al., 2011), *Betula platyphylla* CYP716A180 (Zhou et al., 2016), and *Vitis vinifera* CYP716A15 and CYP716A17 (Fukushima et al., 2011; Figure 5). Other CYP716 family enzymes showing diverse oxidation activities against pentacyclic triterpenoids are C-3 oxidase CYP716A14v2 from *Artemisia annua* (Moses et al., 2015a), C-16α hydroxylase CYP716Y1 from *Bupleurum falcatum* (Moses et al., 2014), and C-22α hydroxylase CYP716A2 from *A. thaliana* (Yasumoto et al., 2016). Additionally, C-12(β) hydroxylase CYP716U1 (CYP716A47) (Han et al., 2011) and C-6(α) hydroxylase CYP716S1v2 (CYP716A53v2) (Han et al., 2012) were reported in ginsenoside (dammarane-type triterpene) biosynthesis in *P. ginseng*. In the present study, we identified CYP716E26 as a novel C-6β hydroxylase against α-amyrin and β-amyrin (Figure 2). This unique enzyme is closely related to tomato CYP716E25 (60.5% identity and 78.7% similarity to CYP716E26) that has not shown any activity in the present study, and *P. ginseng* CYP716S1v2 (CYP716A53v2) shares 49.2% identity and 68.1% similarity to CYP716E26 (Figure 5). It is interesting that these two phylogenetically related P450 enzymes oxidize the same position (C-6) but different types of skeletons (oleanane and dammarane) and stereo-specificity (β and α). Compared with the CYP716A subfamily, in which the C-28 oxidation is the predominant triterpenoid oxidation activity, the subfamilies CYP716E, CYP716S, CYP716U, and CYP716Y catalyzed diverse triterpenoid oxidation reactions. These unique oxidation activities might have developed during their enzymatic evolution from the common ancestor of the CYP716 family enzymes.

### Distribution of triterpenoids in tomato

We determined the contents of triterpenoid aglycones (α-amyrin, β-amyrin, ursolic acid, oleanolic acid, and daturadiol) in tomato leaves, stems, flowers, and roots. Since triterpenoids are often glycosylated in plants, we saponified the tomato extracts before conducting the GC-MS analysis. We could not detect the presence of α-amyrin in any sample; however, lupeol was detected in all analyzed samples (Figure 4A, Supplementary Figure 2). Although, no lupeol producing OSC was reported in tomato, this result indicates the presence of *lupeol synthase* in this plant. Until now, two *OSCs, TTS1*, and *TTS2*, were functionally characterized in tomato (Wang et al., 2011). TTS1 expressed in heterologous yeast system produced only β-amyrin. On the other hand, TTS2 produced δ-amyrin as a major product with six other compounds including β-amyrin, α-amyrin, multiflorenol, ψ-taraxasterol, and taraxasterol. Even though both *OSCs* are expressed in roots (Figure 3), β-amyrin could not be detected in this tissue. Therefore, we speculated that β-amyrin produced by TTS1 and TTS2 was further metabolized in roots. Small peaks corresponding to oleanolic acid were detected in roots and flowers in SIM chromatogram (Figure 4B). In roots, since the expression level of CYP716A46 was under detectable levels, CYP716A44, which is strongly expressed, is supposed to oxidize β-amyrin into oleanolic acid (Figure 3). In flowers, oleanolic acid might be biosynthesized through the activity of CYP716A44 and/or CYP716A46 from β-amyrin, because both *C-28 oxidases* were weakly expressed in this organ (Figure 3). Additionally, even though we could not detect α-amyrin, its C-28 oxidized product ursolic acid was detected in roots (Figure 4). This is also supposed to be the product of the oxidation reaction catalyzed by CYP716A44. From those observations, we concluded that CYP716A44 is involved in both ursolic acid and oleanolic acid biosynthesis in tomato roots. In contrast, even though CYP716E26 was highly expressed in roots, C-6β oxidized triterpenoid, daturadiol, was not detected. There are two possible hypotheses to explain this phenomenon. One is that CYP716E26 has weaker oxidation activity or lower expression level than those of CYP716A44, and the other is that C-6β oxidized triterpenoid was further metabolized in roots. Moreover, Kemen and co-workers have recently reported that accumulation of elevated level of β-amyrin in roots induces the root phenotypic change in oat (*Avena strigosa*; Kemen et al., 2014). The specific expression of triterpenoid oxidases, *CYP716A44* and *CYP716E26* in roots, might have physiological functions *in planta*. To reveal the roles of *CYP716A44, CYP716E26*, and triterpenoids such as β-amyrin and oleanolic acid *in planta*, generation of its correspondent overexpression and knockout tomato lines will be required. Because the mutations are randomly introduced into the genome, it was difficult to generate the objective mutant plants for a long time. Recently, a genome-editing technology, which utilizes the site-specific nucleases, such as TALEN and CRISPR-Cas9, was developed and applied to plants, including tomato (Brooks et al., 2014; Lor et al., 2014). This powerful technology can easily induce the targeted mutagenesis in the plant genome. The generation of the *cyp716-*knockout tomato plants will provide us with useful information about the triterpenoid biosynthesis in plants, and it is a subject of future research in our laboratory.

## Author contributions

SY and YS performed the experiments. SY wrote the manuscript. HS, EOF, and TM supervised the research.

## Funding

This study was partially supported by a Grant-in-Aid for the Japan Society for the Promotion of Science Fellows to SY and a Grant-in-Aid for Scientific Research nos. 23108513 to HS and JP15H04485 to TM.

### Conflict of interest statement

The authors declare that the research was conducted in the absence of any commercial or financial relationships that could be construed as a potential conflict of interest.

## Supplementary material

Supplementary Figure 1 **NMR spectra of compound 8, daturadiol. (A)** ^1^H and **(B)** ^13^C NMR signals.

Supplementary Figure 2 **Mass fragmentation patterns**. Mass spectra of lupeol, β-amyrin, and ursolic acid detected in extracts from Micro-Tom are shown.

Supplementary Table 1 **Primers used in the present study**.

Supplementary Table 2 **Yeast strains used in the present study**.

Supplementary Table 3 **^**13**^C NMR spectra data of compound 8 (daturadiol)**.

## Abbreviations

GC-MS: gas chromatography-mass spectrometry
NMR: nuclear magnetic resonance
OSC: oxidosqualene cyclase
P450: cytochrome P450 monooxygenase
qPCR: quantitative real-time polymerase chain reaction
RT: retention time
SIM: selective ion monitoring
TMS: trimethylsilyl.
