# Supplementary figures and images for: Functional Characterization of CYP716 Family P450 Enzymes in Triterpenoid Biosynthesis in Tomato
Source: Front Plant Sci. 2017 Jan 30;8:21. doi: 10.3389/fpls.2017.00021 (PMC5278499; doi:10.3389/fpls.2017.00021)

(A)

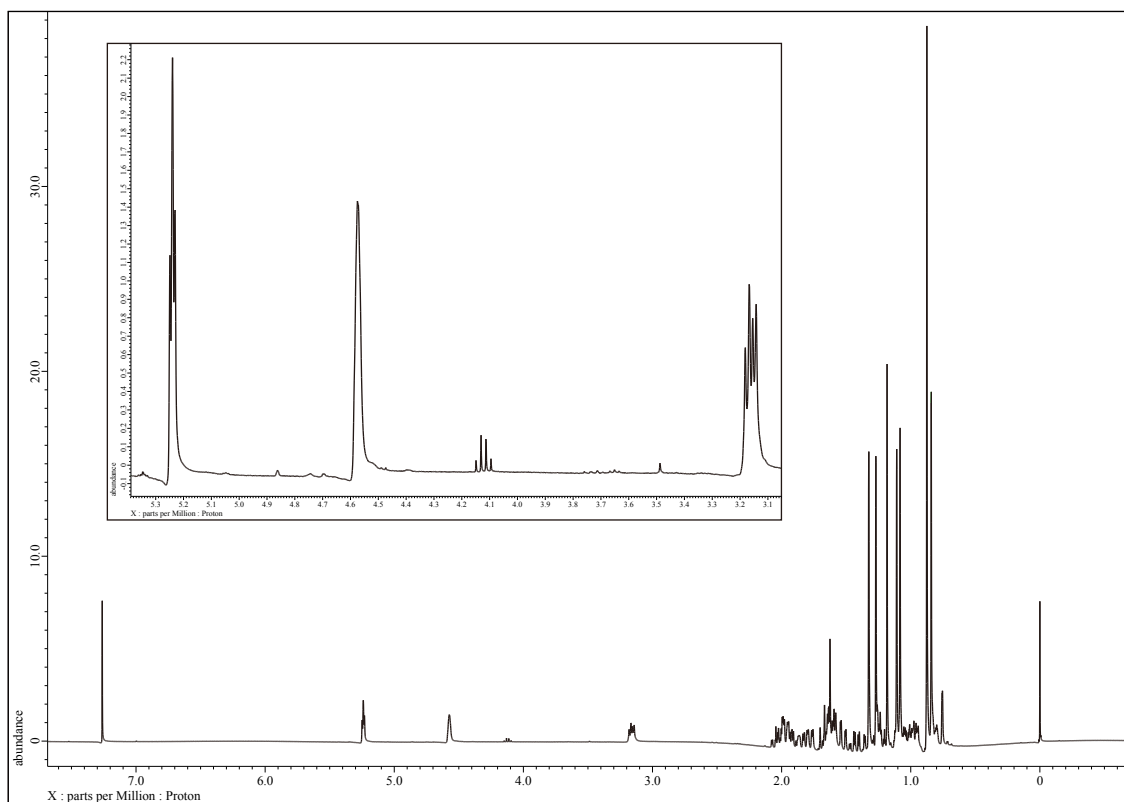

(B)

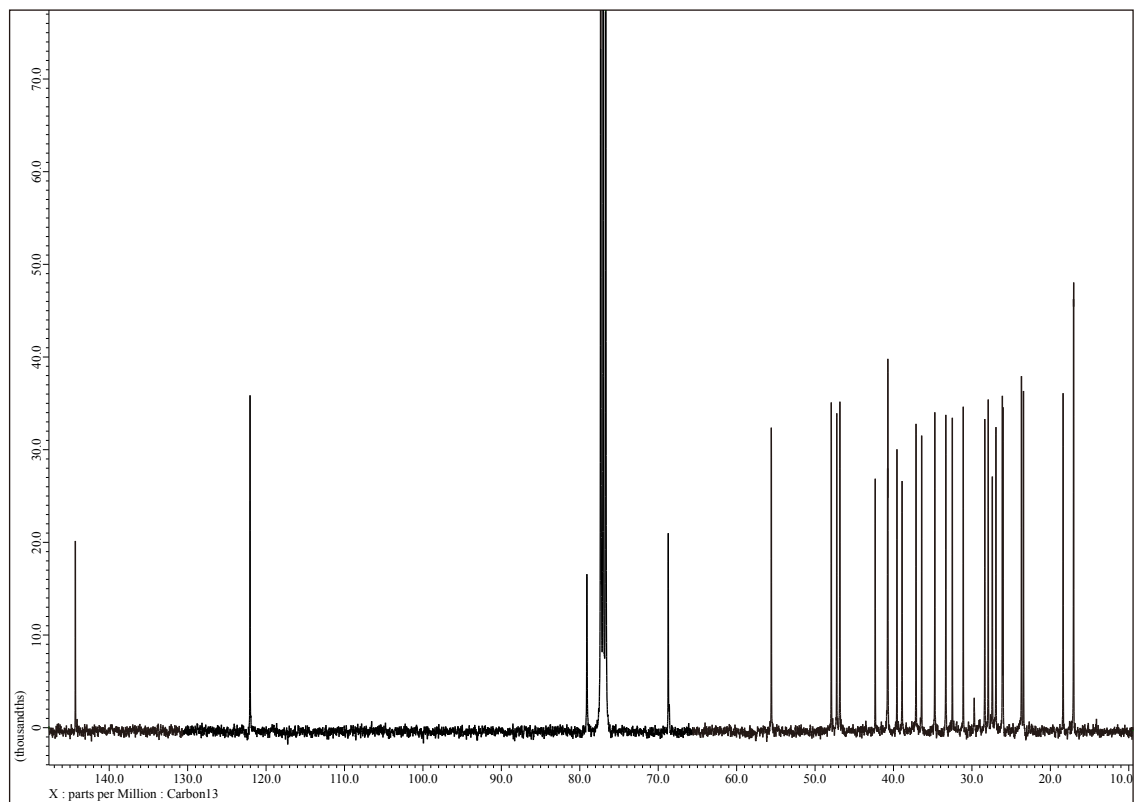

Supplement: Supplementary Figure 1 — NMR spectra of compound 8, daturadiol. (A) 1H and (B) 13C NMR signals. [file Image1.PDF]

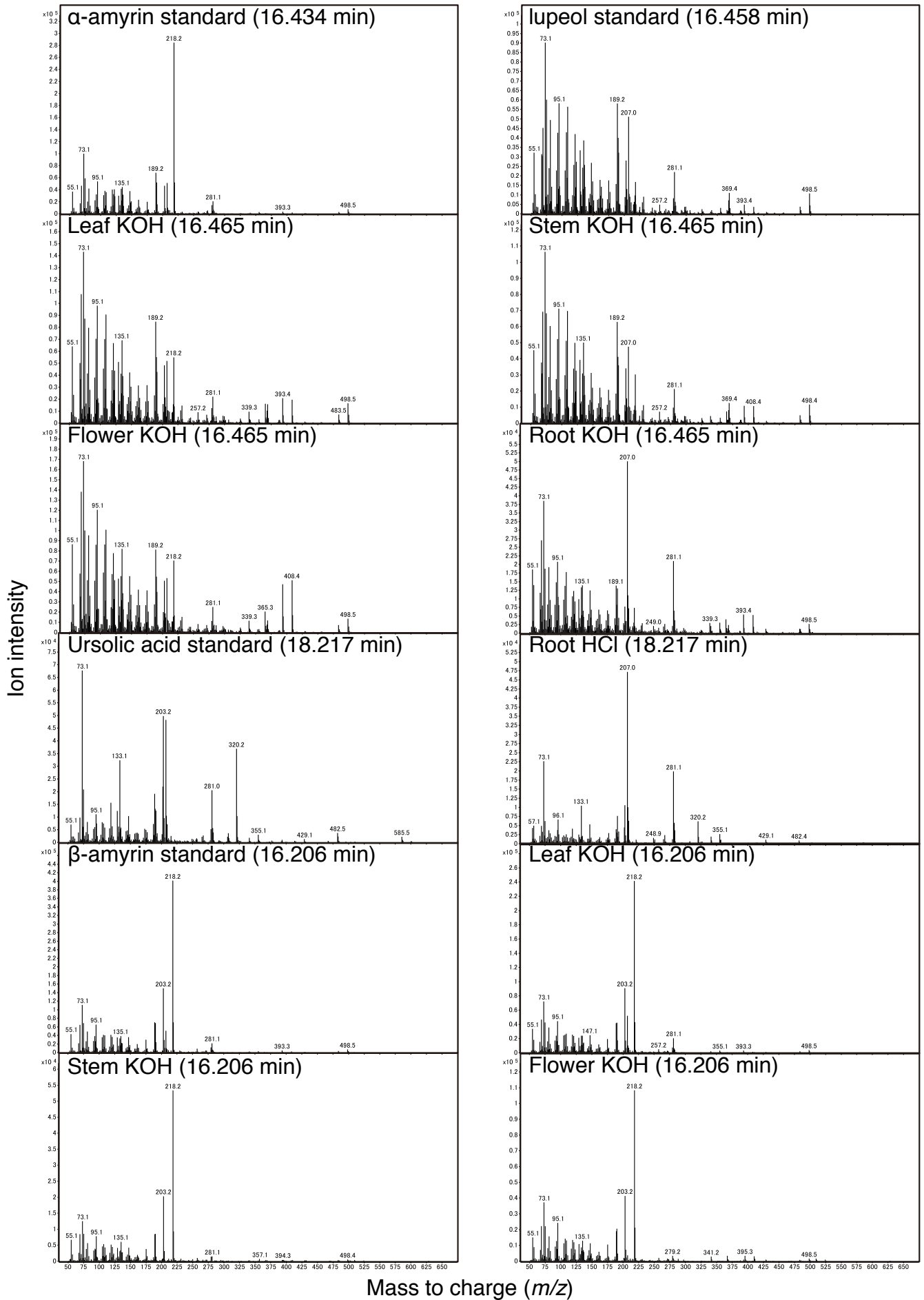

Supplement: Supplementary Figure 2 — Mass fragmentation patterns. Mass spectra of lupeol, β-amyrin, and ursolic acid detected in extracts from Micro-Tom are shown. [file Image2.PDF]
