# Supplementary material for: Functional Characterization of CYP716 Family P450 Enzymes in Triterpenoid Biosynthesis in Tomato
Source: Front Plant Sci. 2017 Jan 30;8:21. doi: 10.3389/fpls.2017.00021 (PMC5278499; doi:10.3389/fpls.2017.00021)
Supplement: Supplementary Table 1 — Primers used in the present study. [file Table1.PDF]

Supplementary Table 1. Primers used in the present study.

Primer	Sequence (5'→3')	Details
1343/CYP716A44-For	caccATGGAGTTGTTGTATGTCTGTCTTG	cloning (CYP716A44)
1344/CYP716A44-Rev	TCACGGGTTGTGGTGGTGAGGGTAGAGTC	cloning (CYP716A44)
1345/CYP716H1-For	caccA TGGACCTCTTCCTCCTCTCTTTTGCAA	cloning (CYP716H1)
1346/CYP716H1-Rev	TTAAATAGAAGAAAGGGTAATAGGATACCCATC	cloning (CYP716H1)
pY178/attB1-AP2	GGGGACAAGTTTGTACAAAAAAGCAGGCTCCGCGGCCGCCCCCTT	adapter primer for attB1
pY179/attB2-AP2	GGGGACCACTTTGTACAAGAAAGCTGGGTCGGCGCGCCCACCCTT	adapter primer for attB2
pY319/SlCYP716_1_For	CACCATGGAATTGTTCTATGCCTCTCTTGT	cloning (CYP716A46)
pY320/SlCYP716_1_Rev	TTAAGTTTTAACGTGATGAGGATAGAGTCT	cloning (CYP716A46)
pY321/SlCYP716_2_For	CACCATGGATCCCTTTATTCTTTATTCACTAGCA	cloning (CYP716E26)
pY322/SlCYP716_2_Rev	TCAAGCTATGGGTTGAATCCTAACA	cloning (CYP716E26)
pY323/SlCYP716_3_For	CACCATGGATGCTATTGATCTCTCCACC	cloning (CYP716C6)
pY324/SlCYP716_3_Rev	TTACTGATGATGGTGAAGACGAATGG	cloning (CYP716C6)
pY325/SlCYP716A42-F	CACCATGGAAGTAGTTATGGTTCCTTACCTATTAG	cloning (CYP716E25)
pY326/SlCYP716A42-R	TTAATTTCCATGAGGCAGAAGGCG	cloning (CYP716E25)
pY327/CYP716A43-F2	CCGCGGCCGCCCCCTTCACCATGGATCCCTTTATTCTTTAT	cloning (CYP716E26)
pY328/CYP716A43-R2	CGGCGCGCCCACCCTTTCAAGCTATGGGTTGAATCCTAACA	cloning (CYP716E26)
pY337/A46-1Ex-Rev	AATCTCTATTTGCTCTTTGTAAACTTGATCATAAATTTC	cloning (CYP716A46)
pY338/A46-2Ex-For	AAGTTTACAAAGAGCAAATAGAGATTGCAAAATCAAAGG	cloning (CYP716A46)
pY339/A46-2Ex-Rev	GCACTCCAATATATCTTCCATCCTTTTGGAATGGAGAA	cloning (CYP716A46)
pY340/A46-3Ex-For	AAGGATGGAAGATATATTGGAGTGCAAATTCAACACA	cloning (CYP716A46)
pY341/716A46-F2	CCGCGGCCGCCCCCTTCACCATGGAATTGTTCTATGCC	cloning (CYP716A46)
pY342/716A46-R2	CGGCGCGCCCACCCTTTTAAGTTTTAACGTGATGAGGA	cloning (CYP716A46)
pY369/A42-qRT-F2	GTTTCACAATTCCAAAAGGGTGGA	qRT-PCR (CYP716E25)
pY370/A42-qRT-R2	TCTTGAAGGATCGAACTTTTCTGGA	qRT-PCR (CYP716E25)
pY373/A43-qRT-F2	TAATGCCACCAGCTCAAGGT	qRT-PCR (CYP716E26)
pY374/A43-qRT-R2	GGTCAAATTTTTCTGGCTCTGGAA	qRT-PCR (CYP716E26)
pY377/A44-qRT-F2	GGGAAGCCCTTTCTGATTTCATG	qRT-PCR (CYP716A44)
pY378/A44-qRT-R2	GGTCCGCTTCCTTCAAATCTTGAT	qRT-PCR (CYP716A44)
pY379/A46-qRT-F1	GTTAAATACCTTGCTGAGCTTCCTG	qRT-PCR (CYP716A46)
pY380/A46-qRT-R1	CAACTCAATAATTCTCCTGGACCCT	qRT-PCR (CYP716A46)
pY383/C6-qRT-F1	TTGCAACAACTATAACTTTCCTCGTG	qRT-PCR (CYP716C6)
pY384/C6-qRT-R1	CTTTGTCGCCGCAATCTCCTT	qRT-PCR (CYP716C6)
pY387/H1-qRT-F1	GAAGTACCTTCAACAAATGCCTGAG	qRT-PCR (CYP716H1)
pY388/H1-qRT-R1	CTTTCCTTGGCATCATTTGCCTT	qRT-PCR (CYP716H1)
pY395/TTS1-F2	TGGCGGTTTAGCAGCATGG	qRT-PCR (TTS1)
pY396/TTS1-R2	AGGCAGTGCATTCGACGTAC	qRT-PCR (TTS1)
pY397/TTS2-F2	TGGGGGTTTATCAGCGTGG	qRT-PCR (TTS2)
pY398/TTS2-R2	CGAGCTAGTGCACTCAACATGC	qRT-PCR (TTS2)
pY399/SlAct-F1	CACCATTGGGTCTGAGCGAT	qRT-PCR (actin)
pY400/SlAct-R1	GGGCGACAACCTTGATCTTC	qRT-PCR (actin)
