# Supplementary material for: Functional Characterization of CYP716 Family P450 Enzymes in Triterpenoid Biosynthesis in Tomato
Source: Front Plant Sci. 2017 Jan 30;8:21. doi: 10.3389/fpls.2017.00021 (PMC5278499; doi:10.3389/fpls.2017.00021)
Supplement: Supplementary Table 2 — Yeast strains used in the present study. [file Table2.PDF]

Supplementary Table 2. Yeast strains used in the present study.

| Strain | Genotype |
|---|---|
| INV*Sc*1 | *MATa his3D1 leu2 trp1-289 ura3-52 MATα his3D1 leu2 trp1-289 ura3-52* |
| SY27 | INV*Sc*1; pYES3[*ADH*/*aAS*], pESC-LEU[*GAL10*/*CPR*], pYES2/CT, pESC-HIS |
| SY30 | INV*Sc*1; pYES3[*ADH*/*bAS*], pESC-LEU[*GAL10*/*CPR*], pYES2/CT, pESC-HIS |
| SY50 | INV*Sc*1; pYES3[*ADH*/*aAS*], pESC-LEU[*GAL10*/*CPR*, *GAL1*/*CYP716E25*], pYES-DEST52[*GAL1*/*CYP716E25*], pESC-HIS[*GAL1*/*CYP716E25*] |
| SY51 | INV*Sc*1; pYES3[*ADH*/*aAS*], pESC-LEU[*GAL10*/*CPR*, *GAL1*/*CYP716E26*], pYES-DEST52[*GAL1*/*CYP716E26*], pESC-HIS[*GAL1*/*CYP716E26*] |
| SY52 | INV*Sc*1; pYES3[*ADH*/*aAS*], pESC-LEU[*GAL10*/*CPR*, *GAL1*/*CYP716A44*], pYES-DEST52[*GAL1*/*CYP716A44*], pESC-HIS[*GAL1*/*CYP716A44*] |
| SY53 | INV*Sc*1; pYES3[*ADH*/*aAS*], pESC-LEU[*GAL10*/*CPR*, *GAL1*/*CYP716C6*], pYES-DEST52[*GAL1*/*CYP716C6*], pESC-HIS[*GAL1*/*CYP716C6*] |
| SY54 | INV*Sc*1; pYES3[*ADH*/*aAS*], pESC-LEU[*GAL10*/*CPR*, *GAL1*/*CYP716H1*], pYES-DEST52[*GAL1*/*CYP716H1*], pESC-HIS[*GAL1*/*CYP716H1*] |
| SY55 | INV*Sc*1; pYES3[*ADH*/*bAS*], pESC-LEU[*GAL10*/*CPR*, *GAL1*/*CYP716E25*], pYES-DEST52[*GAL1*/*CYP716E25*], pESC-HIS[*GAL1*/*CYP716E25*] |
| SY56 | INV*Sc*1; pYES3[*ADH*/*bAS*], pESC-LEU[*GAL10*/*CPR*, *GAL1*/*CYP716E26*], pYES-DEST52[*GAL1*/*CYP716E26*], pESC-HIS[*GAL1*/*CYP716E26*] |
| SY57 | INV*Sc*1; pYES3[*ADH*/*bAS*], pESC-LEU[*GAL10*/*CPR*, *GAL1*/*CYP716A44*], pYES-DEST52[*GAL1*/*CYP716A44*], pESC-HIS[*GAL1*/*CYP716A44*] |
| SY58 | INV*Sc*1; pYES3[*ADH*/*bAS*], pESC-LEU[*GAL10*/*CPR*, *GAL1*/*CYP716C6*], pYES-DEST52[*GAL1*/*CYP716C6*], pESC-HIS[*GAL1*/*CYP716C6*] |
| SY59 | INV*Sc*1; pYES3[*ADH*/*bAS*], pESC-LEU[*GAL10*/*CPR*, *GAL1*/*CYP716H1*], pYES-DEST52[*GAL1*/*CYP716H1*], pESC-HIS[*GAL1*/*CYP716H1*] |
| SY65 | INV*Sc*1; pYES3[*ADH*/*aAS*], pESC-LEU[*GAL10*/*CPR*, *GAL1*/*CYP716A46*], pYES-DEST52[*GAL1*/*CYP716A46*], pESC-HIS[*GAL1*/*CYP716A46*] |
| SY66 | INV*Sc*1; pYES3[*ADH*/*bAS*], pESC-LEU[*GAL10*/*CPR*, *GAL1*/*CYP716A46*], pYES-DEST52[*GAL1*/*CYP716A46*], pESC-HIS[*GAL1*/*CYP716A46*] |
