# Supplementary material for: Functional Characterization of CYP716 Family P450 Enzymes in Triterpenoid Biosynthesis in Tomato
Source: Front Plant Sci. 2017 Jan 30;8:21. doi: 10.3389/fpls.2017.00021 (PMC5278499; doi:10.3389/fpls.2017.00021)
Supplement: Supplementary Table 3 — 13C NMR spectra data of compound 8 (daturadiol). [file Table3.pdf]

Supplementary Table 3. $^{13}$C NMR spectra data of compound **8** (daturadiol).

| Assignment | δ$^{13}$C (ppm) compound **8** purified from yeast strain SY56 | δ$^{13}$C (ppm) daturadiol in reference (Araújo and Chaves (2005)) |
|---|---|---|
| C-1 | 40.72 | 40.7 |
| C-2 | 27.40 | 27.4 |
| C-3 | 79.10 | 79.1 |
| C-4 | 39.56 | 39.6 |
| C-5 | 55.58 | 55.6 |
| C-6 | 68.72 | 68.7 |
| C-7 | 40.76 | 40.8 |
| C-8 | 38.91 | 38.9 |
| C-9 | 47.93 | 47.9 |
| C-10 | 36.42 | 36.4 |
| C-11 | 23.43 | 23.4 |
| C-12 | 122.05 | 122.0 |
| C-13 | 144.32 | 144.3 |
| C-14 | 42.33 | 42.3 |
| C-15 | 26.12 | 26.1 |
| C-16 | 26.95 | 27.0 |
| C-17 | 32.50 | 32.5 |
| C-18 | 47.24 | 47.2 |
| C-19 | 46.84 | 46.8 |
| C-20 | 31.11 | 31.4 |
| C-21 | 34.74 | 34.7 |
| C-22 | 37.13 | 37.1 |
| C-23 | 27.92 | 27.9 |
| C-24 | 17.05 | 17.0 |
| C-25 | 17.03 | 17.0 |
| C-26 | 18.37 | 18.4 |
| C-27 | 26.04 | 26.0 |
| C-28 | 28.37 | 28.3 |
| C-29 | 33.33 | 33.3 |
| C-30 | 23.69 | 23.7 |
